# Zoonotic transmission of asymptomatic carriage Staphylococcus aureus on dairy farms in Canterbury, New Zealand

**DOI:** 10.1099/mgen.0.001318

**Published:** 2024-12-04

**Authors:** Christina Straub, William Taylor, Nigel P. French, David R. Murdoch, Patricia Priest, Trevor Anderson, Pippa Scott

**Affiliations:** 1 The Institute of Environmental Science and Research, Auckland, New Zealand; 2 Genomics Aotearoa, Dunedin, New Zealand; 3 Centre for Microbiology and Environmental Systems Science, University of Vienna, Vienna, Austria; 4 The Institute of Environmental Science and Research, Christchurch, New Zealand; 5 Tāwharau Ora, School of Veterinary Science, Massey University, Palmerston North, New Zealand; 6 Department of Pathology and Biomedical Science, University of Otago, Christchurch, New Zealand; 7 Department of Preventive and Social Medicine, University of Otago, Dunedin, New Zealand; 8 Microbiology Department, Canterbury Health Laboratories, Te Whatu Ora – Health New Zealand Waitaha, Christchurch, New Zealand

**Keywords:** AMR, cluster, mastitis, *Staphylococcus aureus*, transmission, virulence

## Abstract

Zoonotic pathogen transmission is of growing concern globally, with agricultural intensification facilitating interactions between humans, livestock and wild animals. *Staphylococcus aureus* is a major human pathogen, but it also causes mastitis in dairy cattle, leading to an economic burden on the dairy industry. Here, we investigated transmission within and between cattle and humans, including potential zoonotic transmission of *S. aureus* isolated from cattle and humans from three dairy farms and an associated primary school in New Zealand. Nasal swabs (*N*=170) were taken from healthy humans. Inguinal and combined nasal/inguinal swabs were taken from healthy cattle (*N*=1163). Whole-genome sequencing was performed for 96 *S*. *aureus* isolates (44 human and 52 cattle). Multilocus sequence typing and assessments of antimicrobial resistance and virulence were carried out. Potential within- and across-species transmission events were determined based on single nucleotide polymorphisms (SNPs). Thirteen potential transmission clusters were detected, with 12 clusters restricted to within-species and one potential zoonotic transmission cluster (ST5). Potential transmission among cattle was mostly limited to single age groups, likely because different age groups are managed separately on farms. While the prevalence of antimicrobial resistance (AMR) was low among both bovine and human isolates, the discovery of an extended-spectrum beta-lactamase gene (*bla*_TEM-116_) in a bovine isolate was concerning. This study provides evidence around frequency and patterns of potential transmission of * S. aureus* on dairy farms and highlights the AMR and virulence profile of asymptomatic carriage *S. aureus* isolates.

**Impact Statement**

The intensification of food-animal production and routine use of antibiotics facilitate the emergence and the spread of zoonotic antibiotic-resistant pathogens. *Staphylococcus aureus* infections are not only an economic burden to the dairy industry but also of public health concern. We investigated the potential for zoonotic transmission, antimicrobial resistance and virulence profiles of *S. aureus* from asymptomatic carriage in humans and cattle in dairy farms in Canterbury, New Zealand. Our research identified one potential zoonotic transmission event, while all other transmission events were clusters identified within species. Key transmission points were (1) between animals in the same herd, (2) between adult cattle and newborn calves and among newborn calves managed together and (3) movement of cattle between management groups. While the overall presence of antimicrobial resistance genes was low, of concern was the discovery of an extended-spectrum beta-lactamase gene (*bla*_TEM-116_) in a bovine isolate.

## Data Summary

Sequencing reads have been deposited in the National Center for Biotechnology Information SRA database under BioProject number PRJNA1044741. Supporting data, code and protocols have been provided within the Methods or as Supplementary Material and can be found on github (https://github.com/chrstraub/Zoonot_transmission_Saureus).

## Introduction

Intensification of agriculture is a significant driver of zoonotic transmission [1]. As agricultural practices undergo intensification to meet the demands of a growing human population, the expansion of livestock farming and increased human–animal interactions create conditions that facilitate the emergence and transmission of zoonotic pathogens [2]. Close contact between animals and humans can drive host-switching events, where a pathogen colonizes a new host followed by subsequent adaptation through acquisition or loss of mobile genetic elements or mutations [3].

*Staphylococcus aureus* is commonly found as a commensal on the skin and mucosa of animals and humans [4] and is also known for its ability to colonize and cause disease in a diverse range of animal hosts and humans [3,5]. The anterior nares of the nose are the most frequent asymptomatic carriage site, followed by skin and other mucosal sites [4]. About one-third of the human population are persistent asymptomatic *S. aureus* nasal carriers [6,8], with <10% harbouring methicillin-resistant *S. aureus* (MRSA) [9,10]. Carriage is a known risk factor for acquiring subsequent infection, which in general can range from superficial skin and soft tissue infections to invasive infections, such as bacteraemia or osteomyelitis [11,12].

Healthy bovines can also be colonized by pathogenic lineages associated with mastitis [13]; however, colonization rates in the nasal cavity are lower (5–15%) than in humans [14,16]. Bovine mastitis caused by *S. aureus* is a particular problem in the dairy industry affecting animal health and is continuing to cause significant economic losses in New Zealand and worldwide [17,18]. The spread of *S. aureus* to humans from livestock is a well-documented zoonotic event with host switching being recorded among wild animals, goats, cattle, sheep, horses, pigs and domestic pets [3]. The highest rate of zoonotic transmission has been recorded for veterinarians and farm workers, indicating that proximity and prolonged contact with *S. aureus* carriers or infected animals is a greater contributor [19].

The emergence of antibiotic resistance via horizontal gene transfer in *S. aureus* is a serious public health threat, as even without antibiotic resistance, it can cause deadly and difficult-to-treat illness [20]. Of particular concern is MRSA. While MRSA previously was primarily associated with the healthcare setting, it is now known that infections occur in the community and there have been cases of livestock-associated MRSA [21,22]. Increased risk of carriage has been associated with people in close contact with livestock, and livestock density has been shown to be a high-risk factor for the prevalence of carriage of livestock-associated MRSA (LA-MRSA) [23].

Using whole-genome sequencing (WGS), our aim was to determine (1) AMR and virulence profiles of asymptomatic carriage * S. aureus* bovine and human isolates and (2) possible transmission events between and among species, as well as among different cattle age groups in a dairy farming community in New Zealand.

## Methods

### Sample collection

For this study, *S. aureus* isolates were collected from a dairy farming community in Canterbury. Samples were taken from cattle and humans living and/or working on three different dairy farms on non-contiguous land in rural Canterbury during three sampling rounds. Additionally, samples were collected from children and staff from the local (rural) primary school at each of the three rounds. Round 1 was done in the middle of the milking period in February/March 2019, round 2 in June/July 2019 during the dry cow period, where cattle were not being milked, and round 3 in August/September 2019 during calving.

#### Bovine isolates

**Fig. 1.**
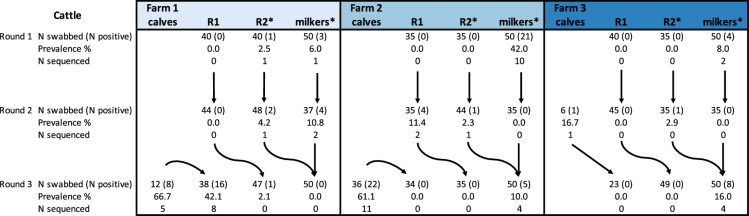
Overview of collected bovine samples. Number (*N*) of samples that were collected and tested positive for *S. aureus* per farm, sampling round and age group. R2s and milkers can have calves. Arrows indicate the shift of cattle between age groups and herds.

On each farm, the three age groups R1 (0–12 months old), R2 (13–24 months old) and adult cows (>24 months) were sampled three times (Fig. 1). The age groups were managed separately on each farm (i.e. not on the same pasture at the same time, but may have been on adjacent land to other groups on the same farm and had shared husbandry facilities). In round 3, newborn calves were housed separately, under shelter in pens shared with a small number of other calves. The slightly older, still milk-fed calves (R1s) were on pasture. The newborns were merged with R1s as they became old enough. Newborns were sampled separately from R1s as they were likely to have different contacts and epidemiology.

Approximately 50 animals were sampled from each group on each farm at each round. Lactating cows were sampled on rotary milking platforms, whereas R1s, R2s and dry cows (adult cows not currently producing milk) were sampled in races while other procedures, such as worming or vaccination, were being undertaken. True random selection was challenging, given the time available to sample each individual (e.g. on the rotary milker, only 10 s was available to identify and sample each individual before they moved past the sampling stand), so every *N*th animal was sampled (where *N* is group size/the number of intended swabs).

Lactating cows and R2s had swabs taken from the inguinal skin (between udder and leg) using a sterile, gel-moistened swab (COPAN Transystem, Amies transport media). This was performed instead of nasal swabs because of the difficulty of access to the nasal area in the management facilities. In rounds 1 and 2, R1s had swabs were taken from inguinal skin; however, in round 3, newborn and older calves had combined nasal and inguinal swabs taken wherever possible.

#### Human isolates

All individuals working or living on each of the three farms or children attending the primary school were asked to participate in the study. Those who provided informed consent underwent sampling for nasal carriage of *S. aureus* at each sampling round. Swabs were taken from the anterior nares using a sterile, gel-moistened swab (COPAN Transystem, Amies transport media). Both nostrils were sampled using the same swab. No participants reported infected skin lesions at any study round.

### Transport, culturing and DNA extraction

Swabs were processed at Canterbury Health Laboratories, Christchurch. Samples were enriched by incubation at 35 °C in a brain–heart infusion-based broth containing mannitol, colistin and aztreonam and, after a minimum of 16 h, streaked onto CHROMagar *S. aureus* media (Fort Richard, Auckland, New Zealand). The next day, plates were checked for the growth of pink colonies indicative of *S. aureus*. The identity of *S. aureus* was confirmed using a coagulase test and by MALDI-TOF mass spectrometry (Bruker Daltonics). A subset of 96 isolates were chosen for WGS as follows: for isolates from cattle, half of the isolates from each age group on each farm were selected by taking every second isolate (by order of collection from cattle). For human isolates, the first *S. aureus* isolate obtained from each individual was sequenced, with a small number of multiple isolates from the same individual having been sequenced to provide information on the number of SNPs between sequential isolates from the same source. Each stored *S. aureus* isolate was cultured and pre-treated with Lysostaphin (Sigma-Aldrich) before DNA was extracted from the GenElute Bacterial Genomic DNA Kit (Sigma-Aldrich) following the manufacturer’s protocol. DNA quality was checked using gel diffusion and the Qubit broad-range dsDNA Quantitation kit (Thermo Fisher Scientific).

### WGS, assembly and quality control

Sequencing libraries were prepared using the NEBNext Ultra DNA Library Prep Kit (New England Biolabs), and sequencing was performed using an Illumina NovaSeq 6000 with 2×150 bp paired-end reads (Novogene). Raw reads from Illumina sequencing were quality-checked, and adapter sequences were trimmed with Trimmomatic v.0.39 [24]. Reads were assembled using skesa v.2.3.0 [25] integrated in the nullarbor2 pipeline (https://github.com/tseemann/nullarbor). Assembly quality was assessed using Quast v.5.0.2 [26]. Due to a large number of contigs, the genomes of three isolates (B123-028, B323-3115, H1121-016) were re-assembled using downsampling of the sequencing data to 100× coverage using rasusa v.0.7.0 (https://github.com/mbhall88/rasusa) and quality trimming using trimmomatic, which improved the quality of the genomes. Two genome assemblies (B213-1014 and H1121-016) with >150 contigs or an unusual genome size >3 Mb were removed from downstream analysis. Kraken v.1.1.1 was used to classify reads from each isolate to confirm species identity. The reference strains used for analysis were *S. aureus* NCTC 8325 (ST8, accession number GCF_000013425.1), MSSA476 (ST1, GCF_000011525.1), UCI28 (ST5, NZ_CP018768.1), ILRI_Eymole1/1 (ST30, NZ_LN626917.1) and NCTC9553 (ST151, UHCG00000000.1). Multilocus sequence typing was performed using multilocus sequence typing v.2.19.0 (https://github.com/tseemann/mlst). Novel alleles and sequence types (STs) not present in the database were submitted to pubMLST (https://pubmlst.org/) to obtain allele and ST assignments.

### AMR and virulence finder

Draft assemblies were screened for genetic determinants of antimicrobial resistance using abriTAMR v.1.0.13 (https://github.com/MDU-PHL/abritamr) and the AMRFinderPlus database (09 August 2022). ABRicate v0.9.8 (T. Seemann [27]) was used to identify virulence factors based on the virulence factor database [28] and plasmids using the PlasmidFinder database [27]. Screening for staphylococcal cassette chromosome *mec* (SCCmec) elements was done using SCCmecFinder v.1.2. (https://cge.food.dtu.dk/services/SCCmecFinder/). Genomes were further screened for the presence of prophages using the webserver PHASTEST [29].

### Phylogenetic inference

Trimmed reads were aligned to the reference using bwa mem v0.7.17 [30], and duplicate sequences were marked using Picard v2.23.6 [31]. SNPs were called using Freebayes v1.3.2 [32] under a haploid model with a minimum mapping quality of 30, minimum coverage of 10× and a minimum alternate allele fraction of 0.7. Furthermore, SNP filtering was performed using vcftools v0.1.16 [33]. Identical workflows were applied using reference strains *S. aureus* NCTC8325 for all 98 isolates, MSSA476 (ST1) for 42 isolates identified as clonal complex 1 (CC1), UCI28 (ST5) for 13 isolates identified as CC5, ILRI_Eymole1/1 (ST30) for 7 isolates identified as CC30 and NCTC9553 (ST151) for 13 isolates identified as CC151. The resulting whole-genome sequence alignments were processed using gubbins v.3.2.1 [34] to remove recombinant regions.

Prior to building the phylogenies, snp-sites v 2.5.1 [35] was used to extract SNP sites from the recombinant-cleaned gubbins multi-FASTA whole-genome alignment. Iqtree v2.2.0.3 [36,37] was used to build maximum-likelihood (ML) phylogenies using 1000 bootstrap iterations [38].

### Genetic structure and transmission analysis

Pairwise SNP distances between isolates were calculated based on the recombinant-free whole-genome alignment using the dist.gene function (R package ‘ape’ [39]). Hierarchical single-linkage clustering was performed using the hclust function and then filtered based on a SNP cut-off of <14 using the function cutree (R package ‘stats’). To further support the results of the clustering analysis, ska (https://github.com/simonrharris/SKA), a split k-mer analysis, was performed on the whole-genome assemblies to calculate pairwise distances between isolates using k-mers with a length of 15 bp and a threshold of 14 SNPs with 99% identity.

The 14 SNP cut-off was chosen based on the upper limit of the pairwise SNP distance (range 5–14 SNPs) recovered among three pairs of human isolates, each derived from the same individual from two consecutive sampling points during the study period. Our cut-off is more stringent than the maximum genetic distance for transmission of MRSA described by Coll *et al.* [40] with the conservative cut-offs of 25 whole-genome SNPs or 15 core-genome SNPs. Furthermore, looking at the pairwise SNP distributions (Fig. S1, available in the online Supplementary Material) of the individual CC transmission analysis, a 14-SNP cut-off covers the most closely related cases.

## Results

A total of 170 swabs were taken from humans (Table 1) and 1163 swabs from cattle (Fig. 1). *S. aureus* was isolated from 94 (55%) and 102 (8.8%) of these swabs, respectively. The prevalence of asymptomatic *S. aureus* carriage was higher in children (33–100% depending on group and sampling round) than adults (0–70%), but some groups had few participants limiting the precision of these estimates. In cattle, *S. aureus* was detected most frequently in young calves and generally more frequently in adult cows than R1s and R2s (Fig. 1).

**Table 1.** Overview of collected human samples

		Farm 1		Farm 2		Farm 3		School	
		**Children**	**Adults**	**Children**	**Adults**	**Children**	**Adults**	**Children**	**Adults**
**Round 1**	***N* swabbed (*N* positive**)	6 (2)	8 (2)	3 (3)	10 (7)	4 (2)	7 (3)	15 (12)	3 (0)
	**Prevalence %**	33.3	25.0	100.0	70.0	50.0	42.9	80.0	0.0
	***N* sequenced**	2	2	2	6	2	3	12	0
**Round 2**	***N* swabbed (*N* positive**)	6 (3)	10 (2)	3 (3)	9 (6)	4 (3)	9 (6)	12 (9)	3 (0)
	**Prevalence %**	50.0	20.0	100.0	66.7	75.0	66.7	75.0	0.0
	***N* sequenced**	1	0	0	4	2	3	1	0
**Round 3**	***N* swabbed (*N* positive**)	5 (3)	12 (3)	2 (2)	9 (4)	4 (4)	9 (5)	14 (9)	3 (1)
	**Prevalence %**	60.0	25.0	100.0	44.4	100.0	55.6	64.3	33.3
	***N* sequenced**	0	1	0	1	1	1	0	1

Number (*N*) of samples collected and tested positive for *S. aureus* per farm and for the school, sampling round and age group.

WGS was performed on 45 of 94 *S*. *aureus* isolates from human swabs and 53 of 102 cattle swabs. After quality control, 96 isolates (Table S1; 52 cattle and 44 human isolates) underwent genomic analyses. Bovine isolates were from either skin or nose swabs. Out of the 44 human isolates, 30 were collected from individuals living/working at the farm and 14 isolates were collected from pupils and adults at a close-by school.

The genomes had an average of 29 contigs (±22 sd) and a median N50 of 226 409 bp (Table S1). Genome coverage to the *S. aureus* NCTC8325 reference (GCA_000013425.1) was an average of 90.4% (±2.5% sd).

### Typing

#### Multilocus sequence typing

**Fig. 2.**
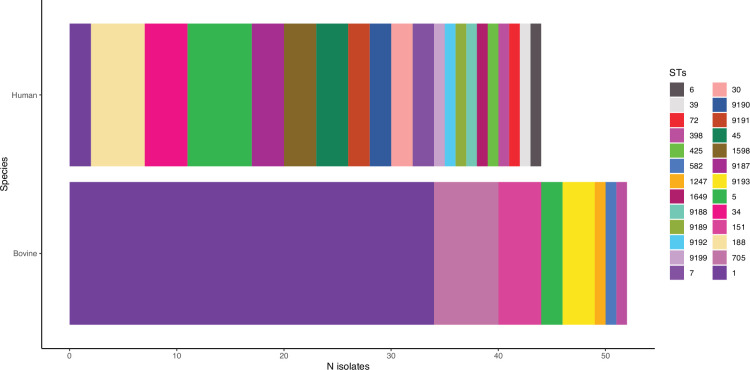
Barplot showing the diversity of STs observed for human and bovine samples. The number of isolates observed for each ST is plotted.

A total of 26 STs were recovered from the dataset, with 8 previously unreported STs identified. For bovine isolates, eight STs were identified, with the most common STs being ST1/CC1 (*N*=34, 63%), ST705/CC151 (*N*=6, 11.1%) and ST151/CC151 (*N*=4, 11.1%) (Fig. 2).

**Fig. 3.**
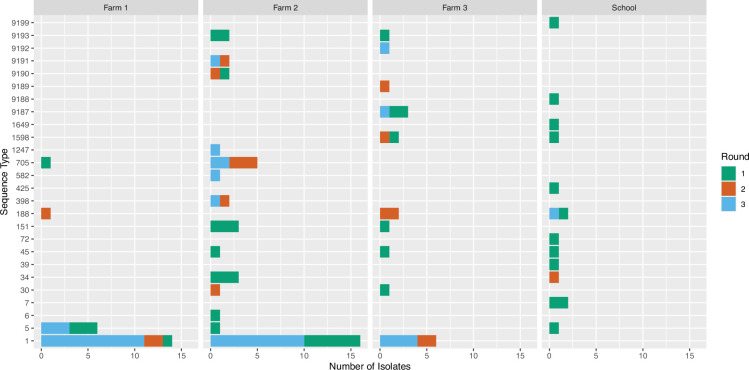
Number of isolates per ST observed for each sampling location (Farms 1–3 and school). The different colours depict each of the three sampling rounds.

Much more diversity was discovered for human isolates, with 21 different STs, and the most common STs were ST5/CC5 (*N*=6, 13.6%), ST188/CC1 (*N*=5, 11.4%) and ST34/CC30 (*N*=4, 9.1%). Only ST1, ST5 and ST398 were found in both species, and ST1 and ST151 were found on all three farms but not in the school setting (Fig. 3). ST1 was found persistently on Farm 1 during all three sampling rounds.

### Antimicrobial resistance

A total of 21 antimicrobial resistance genes were identified for all isolates with an average of six and three AMR genes for human and cattle isolates, respectively. All isolates carried genes associated with resistance to at least two antibiotic classes. The tetracycline resistance gene *tet(38*) and *mepA*, part of the MepRAB cluster encoding an MDR-efflux pump, were found in all isolates. Three isolates had additional *tet* genes (*tet*(*K*), *tet*(*M*)). Penicillin resistance was detected across 40 isolates conferred by the presence of either *bla*Z or *bla*PC1, with the majority being human isolates (95%). Twenty-eight isolates carried three genes (*bla*I, *bla*R1 and either *bla*Z or *bla*PC1). *FosB* encoding for a thiol transferase leading to resistance to fosfomycin was recovered for 25 isolates.

All isolates were positive for lmrS, an efflux protein that is associated with multiple drug resistances, such as linezolid, trimethoprim, florfenicol, chloramphenicol, erythromycin, streptomycin, kanamycin and fusidic acid. Two isolates contained macrolide resistance genes (*ermA* for H2281-2 and *ermT* for B320-2017) and plasmid-encoded aminoglycoside resistance genes (aac(6′)-Ie-aph(2″)-Ia, B320-2017 and *ant(9)-Ia,* H2281-2) conferring the macrolide-lincosamide-streptogramin B (MLSb) phenotype. Two isolates contained the trimethoprim resistance gene *dfrG* (HS11251-421 and H2282-2). Additionally, single isolates carried a broad-spectrum beta-lactamase resistance gene (*bla*_TEM-116_, B123-031), a lincosamide resistance gene (*vgaA*, H2282-2) and a fusidic acid resistance gene (*fusC*, H3391-3).

Three human isolates (HS11251-421, H1322-036 and H2282-2) were positive for methicillin resistance conferred by *mecA. MecA w*as found to be part of the SCCmec type IVc (HS11251-421 and H1322-036) and type Vc (H2282-2). The three *mecA*-positive isolates were assigned different STs (ST30, ST1649 and ST398). Both ST30 and ST1649 were singletons, with only one human isolate in the dataset assigned that ST, with the exception for ST398. One bovine isolate (B320-2017) was also ST398, isolated from the same farm, during different sampling rounds, and it had a distinct AMR profile compared with the human ST398 isolate.

### Biocides and metals

Two isolates contained *qacC* genes associated with resistance to quaternary ammonium compounds used as biocides and disinfectants. A gene associated with cadmium resistance, *cadD,* was identified in 26 isolates; 12 isolates carried the arsenic resistance genes *arsBN*, *arsC* and *arsR*, and six contained the *mco* gene, involved in copper homeostasis and oxidative stress responses.

### Virulence profiles

Isolates were screened for the presence or absence of genes from seven virulence families (enterotoxins, enterotoxin-like proteins, superantigen-like proteins, cytotoxins, adherence, immune evasion and exfoliation). A total of 41 virulence genes were recovered from the isolates with an average of 16 and 14 virulence genes for human and cattle isolates, respectively. Carriage varied with five human isolates carrying ≥20 virulence genes, 87 isolates presenting with ≥10 and four isolates (three human and one cattle) with <10 virulence genes (Table S1).

Five genes were conserved in all 96 isolates, of which three were haemolysin genes (*hld*, *hlgA* and *hlgC*) associated with cattle mastitis. The other two were *aur*, encoding for aureolysin, a conserved protein involved in the proteolytic processing of other staphylococcal proteins, and *icaC*, part of the intercellular adhesion locus *ica,* which is required for biofilm formation. Few isolates carried the major enterotoxin genes *sea* (10/96 isolates), *sec* subtypes 1/2/3 (15/96) and *sed* (3/98), associated with food poisoning.

### Phylogeny

**Fig. 4.**
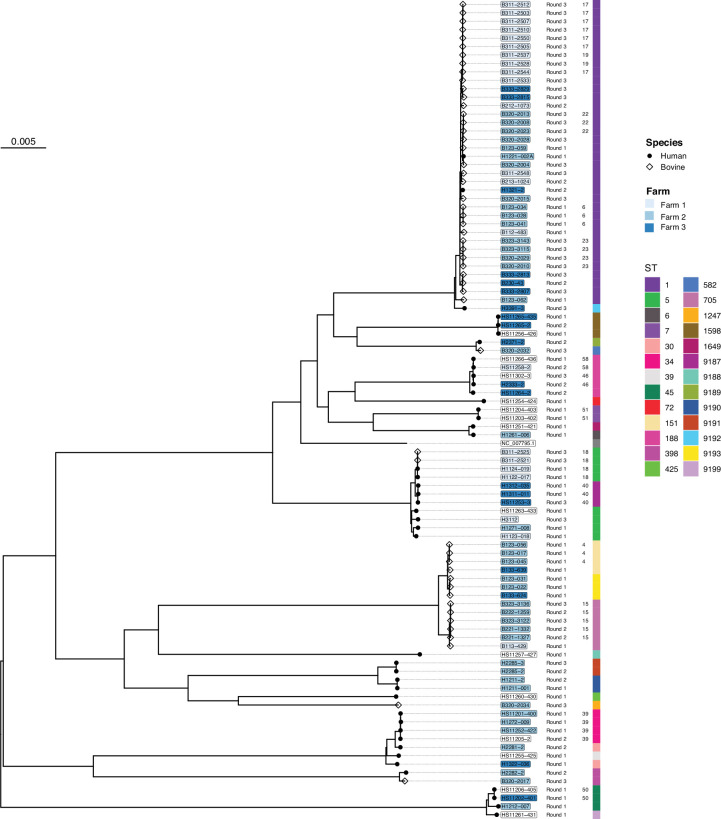
ML phylogeny of 96 bovine and human *S. aureus* isolates based on a recombinant-free alignment of 45 791 polymorphic sites (including 37 663 parsimony informative sites). Isolate source (species) is specified as a symbol at the tip nodes. The tip labels are coloured according to the sampling location (Farms 1 to 3); white bubbles indicate the samples were collected from the school. The sampling round is indicated as text, followed by the transmission cluster ID, and the coloured boxes refer to the STs identified.

**Fig. 5.**
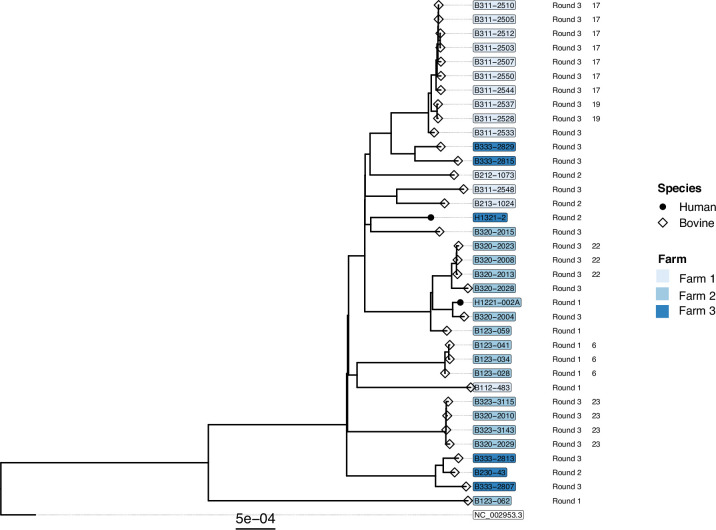
ML phylogeny of 36 *S*. *aureus* isolates classified as ST1, using strain MSSA476 as a reference. The ML tree is based on a recombinant-free alignment of 2082 polymorphic sites (including 1004 parsimony informative sites). The first text column contains sampling round information, followed by transmission cluster ID.

An ML phylogeny was built for all 96 *S. aureus* isolates, and a total of 45 791 polymorphic sites (including 2238 multi-allelic sites) were identified after having removed recombinant regions (Fig. 4). Because of the large genetic distance between the clades, a separate phylogeny was built for all ST1 isolates to provide higher resolution. The ST1 ML phylogeny was based on a whole-genome alignment of 2082 polymorphic sites with two multiallelic sites (Fig. 5).

### Transmission

**Fig. 6.**
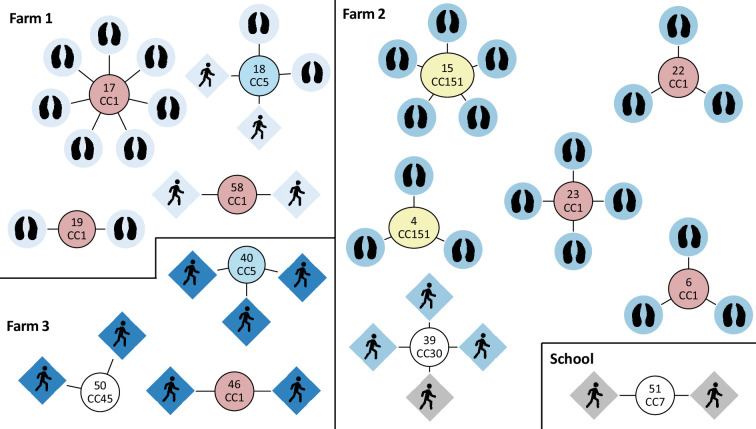
Fourteen potential transmission clusters as identified by using a cut-off threshold of 14 SNPs between isolates. The text circles contain the cluster IDs, as well as the clonal complex that the isolates belong to, and they are colour coded according to CC information. Source of isolation (species) as depicted by symbols, and cluster IDs correlate to those in Table 2. The colour-coded shapes refer to the farm, with grey isolates taken from school children not associated with any of the farms. The number of SNPs for each transmission link can be found in Table S2.

A total of 14 potential transmission clusters were identified that comprised 47% (46/98) of isolates (Table 2, Fig. 6) using a cut-off threshold of 14 SNPs. Details about the number of pairwise SNPs between transmission links can be found in Table S2. Seven of the potential transmission clusters included only bovine isolates, six clusters were human-only isolates, and one cluster was associated with human and bovine isolates. These results do not include three clusters (clusters 34, 45 and 63), which were isolates obtained from the same persons with a 3- to 4-month sampling gap and hence cannot be considered transmission. SNP distances ranged from 5 to 14 SNPs between isolates collected from the same person.

**Table 2.** Transmission cluster metadata. Cluster IDs are indicated with symbols for
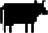
bovine-only,
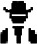
human-only, mixed transmission clusters. ST associated with each transmission cluster, number of isolates in each cluster, isolate IDs, sampling location, sampling round and information regarding age of cattle/humans. Age: 0, calves in shed; 1, R1; 2, R2; 3, adult cows; 4, child; 5, adult

Cluster	ST	*N*	Isolate IDs	Location	Sampling round	Age
4 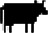	151	3	B123-017, B123-045, B123-056	Farm 2	Round 1	3
6 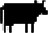	1	3	B123-028, B123-034, B123-041	Farm 2	Round 1	3
15 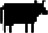	705	5	B221-1327, B221-1332, B222-1259, B323-3122, B323-3136	Farm 2	Round 2,3	1,2,3
17 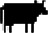	1	7	B311-2503, B311-2505, B311-2507, B311-2510, B311-2512, B311-2544, B311-2550	Farm 1	Round 3	0,1
18 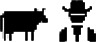	5	4	B311-2521, B311-2525, H1122-017, H1124-019	Farm 1	Round 1,3	1,4,5
19 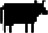	1	3	B311-2528, B311-2537	Farm 1	Round 3	1
22 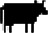	1	3	B320-2008, B320-2013, B320-2023	Farm 2	Round 3	0
23 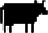	1	4	B320-2010, B320-2029, B323-3115, B323-3143	Farm 2	Round 3	0,3
39 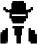	34	4	H1272-009, HS11201-400, HS11205-2, HS11252-422	Farm 2 and school	Round 1,2	4
40 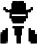	9187	2	H1311-011, H1312-035, HS11253-3	Farm 3	Round 1,3	4,5
46 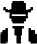	1	2	H2333-2, HS11302-3	Farm 3 and school	Round 2,3	4,5
50 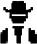	45	2	HS11202-401, HS11206-405	Farm 3 and school	Round 1	4
51 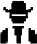	7	2	HS11203-402, HS11204-403	school	Round 1	4
58 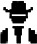	188	2	HS11258-2, HS11266-436	Farm 1 and school	Round 1,2	4

#### Herd transmission

There was evidence of potential transmission between cattle (or a common source of acquisition) on both Farm 1 and Farm 2; however, it was limited to within each farm. Three potential transmission clusters were identified on Farm 1 (clusters 17, 18 and 19). Transmission clusters 17 and 19 contained isolates collected during the third sampling round. With pairwise SNP differences ranging from 7 to 16 SNPs between isolates assigned to either cluster, transmission between animals of either cluster cannot be ruled out. Cattle in cluster 19 were within the same age range (R1), whereas for cluster 17, the potential transmission occurred between newborn calves and R1s. Cluster 18 contains human isolates, so is discussed in the ‘Zoonotic transmission’ section.

Five potential transmission clusters were associated with Farm 2, with most clusters identified for isolates taken during the same sampling round and from cattle of the same age (adult cows in clusters 4 and 6 and newborn calves in cluster 22). A distance of zero SNPs was observed for isolate pairs B123-028 and B123-034 (cluster 6), suggesting very recent transmission between lactating adult cows. Cluster 23 were isolates sampled around calving time and suggested transmission between adult cows and newborn calves. Transmission cluster 15 included isolates obtained from two sampling rounds and cattle of different ages, with R1s and R2s sampled during round 2 and adult cows at round 3, noting that R2s from round 2 had now been merged into the milker herd at round 3.

#### Zoonotic transmission

There was evidence of a potential zoonotic transmission event on Farm 1 with cluster 18 (two human and two bovine isolates). The findings suggested human-to-human transmission (or a common source of acquisition) occurred between parent and child before sampling Round 1. There was no further potential transmission detected to three other sampled members of the household. Subsequently, there was evidence of zoonotic transmission having occurred, with 2 young calves (0 SNPs between isolates from the calves, 7 and 13 SNPs between isolates from humans and the calves and 14 SNPs between the human isolates) at Round 3.

To further investigate the epidemiological link among cluster 18 isolates, isolates were screened for the presence of mobile elements. While no shared plasmids were discovered, an identical 55.4 kb phage was found in all four cluster 18 isolates and identified as a φSa3 prophage (Fig. S2), which integrated into the *β*-haemolysin (*hlb*) gene, with the presence of immune evasion cluster genes *scn* (staphylococcal complement inhibitor), *chp* (chemotaxis inhibitory protein) and *sak* (staphylokinase), whereas *sea* (enterotoxin A) was missing from those four isolates. The φSa3 prophage was also found in all other ST5 isolates in this study showing great variability, with the presence and absence of prophage-encoded genes, which is common for this prophage type (Fig. S2); however, all of the isolates also carried the *scn*/*chp*/*sak* genes.

#### Community transmission

Seven clusters involved only humans, with the detection of potential transmission clusters between pupils at school: an adult and a pair of siblings from Farm 2 and a classmate (cluster 39), a child from Farm 3 and their classmate (cluster 50), a pair of siblings at the school not living on a participating farm (cluster 51), an adult couple and a child on Farm 3 (cluster 40), an adult at school and a child at school/Farm 3 (cluster 46) and two classmates at school (cluster 58).

Split k-mer analysis identified 14 clusters (Table S1), which contained the same isolates as identified with the whole-genome SNP clustering method. Initially, isolate B123-045 was not part of ska cluster 4 due to a 106-SNP distance between B123-045 and B123-056. An investigation revealed the presence of a 42-kb phage in isolates B123-045 and B123-056, which was missing in B123-017. After the removal of the phage, the recalculated ska pairwise SNP distance was 10 SNPs between B123-045 and B123-056. This highlights the importance of using different clustering approaches to confirm results.

## Discussion

In this study, we investigated genetic diversity and carriage of antimicrobial and virulence genes and identified putative zoonotic transmission events. A total of 96 *S. aureus* isolates were collected from cattle and humans from three farms and a local school. All isolates were genotypically resistant to at least two antibiotic classes. Resistance to methicillin (*mecA* gene) was found in three human isolates, but no MRSA was detected among bovine isolates. Putative transmission was mostly limited within cattle management groups (generally age group), with only one potential transmission observed between humans and cattle.

Zoonotic transmission of pathogens is a growing problem as farming intensity and proximity of humans to animals increase [41]. In New Zealand, dairy and beef farming has increased substantially in recent decades, contributing $18.6 billion (5.3% of the gross domestic product) to the New Zealand economy in 2021. The pasture footprint of dairy increased from 1.4 M ha in 1990 to 2.2 M ha [42]. In the same period, dairy cattle numbers increased from 3.4 to 6.1 million. Specifically in the Canterbury region, in which dairy is a major contributor to economic outputs, dairy cattle increased tenfold between 1990 and 2019, from 113 000 to 1.2 million [43]. Additionally, over the last 30 years, the average dairy herd has increased from 135 to >400, while the total number of dairy herds has decreased, indicating intensification in the industry, increasing the potential for the spread of diseases, like close-contact mastitis outbreaks [44].

Most studies in cattle focus on mastitis-causing *S. aureus* typically isolated from milk (e.g. [17,45]), whereas our study represents the diversity of asymptomatic *S. aureus* on inguinal skin by cattle. Previously, it was shown that carriage of *S. aureus* by healthy cows is similar between inguinal region and nares, with a prevalence of pathogenic STs (CC97 and CC133) commonly found in mastitis [13].

In line with our findings, other studies have also found that the prevalence of carriage of *S. aureus* in cattle depended on age group [13,46]. A Japanese study found an overall *S. aureus* carriage rate in cattle of 23.3% [47], while a study of US dairy farms found rates of 35% [48]. In a Swedish study, 11% of dairy cattle were positive for *S. aureus*, while lactating cows had a higher rate of 27%, and the proportion of cattle treated for *S. aureus* mastitis ranged from 8 to 47% [46]. Asymptomatic colonization of nares and udder can be an important source of mastitis and subsequent milk contamination, but little is known about the prevalence and carriage in healthy animals [13,15, 49].

In contrast, anterior nares carriage in humans varies greatly between 20 and 80%, with 20–30% being considered persistent carriers [8,50]. Asymptomatic nasal colonization by *S. aureus* predisposes humans to infection and is considered a major risk factor [50]. The high prevalence of nasal carriage of *S. aureus* seen in children in this study is consistent with findings in our previous study of 53–65% asymptomatic *S. aureus* carriage in primary school-aged children in New Zealand [51] and in studies conducted elsewhere [52,53].

The most common ST found in cattle in our study was ST1 (36/96, 36.7%). In New Zealand, it has been shown that CC1/ST1 has dominated as bovine and mastitis-associated lineage over the past two decades [17,54], whereas in other countries, ST1 associated with bovine isolates is rare [45,55, 56]. We also found ST705, ST151 and ST398, all of these STs are known to be associated with cattle and typically mastitis [18]. Surveys in Canada, Russia and Europe largely identified CC151 and CC97 as the most prevalent clonal complexes in mastitis [45,55, 57].

The genetic diversity of *S. aureus* sampled from cattle (8 STs) and humans (21 STs) in a farm setting is markedly different. Similarly, limited genetic diversity in bovine *S. aureus* was recently shown in studies of milk isolates in China and Canada [57,58], whereas a survey of mastitis milk in southern Xinjiang, China, found a surprising amount of 44 distinct STs among 84 milk samples collected from cattle on two different farms [59].

Overall, 20 genes associated with AMR and 41 virulence genes were identified in this study. The *mecA* gene, associated with resistance to methicillin conferred by the production of penicillin-binding protein (PBP2a), was found in three human isolates (ST30, ST398 and ST1649) but not in any bovine isolate, suggesting that despite the study participants living in an area of high animal density, these isolates are potentially not LA-MRSA. LA-MRSA is very rare in dairy herds in New Zealand [17], but depending on the country, it can be highly prevalent [60]. Whilst we found genotypic evidence of AMR, in particular to tetracycline (tet(38)) and penicillin (*bla*I*, bla*R1*, bla*Z or *bla*PC1), it is important to note that we did not perform phenotypic susceptibility testing to confirm antibiotic resistance, as discussed in the ‘Study limitations’ section.

Interestingly, *blaZ* was present in only 3.7% of bovine isolates in this study compared with 72.7% of human isolates. This is in contrast to 36% penicillin resistance confirmed phenotypically, found in mastitis-causing isolates with most isolates carrying one or more *bla* genes [17]. A strong geographical variability of the presence of *blaZ* associated with mastitis-causing isolates has been reported, with studies from China, Russia or Brazil showing >60% of isolates carry *blaZ* [45,58, 61] compared with 2% in Canada or 14% in Switzerland [56,57].

Extended-spectrum beta-lactamases (ESBL) are the leading cause of resistance to third-generation cephalosporins, and their spread is of major public health concern. While ESBL carriage in *Enterobacteriales* associated with cattle is common globally [62], there are very few reports of ESBL in *S. aureus* [63,64]. The discovery of plasmid-associated broad-spectrum beta-lactamase *bla*_TEM-116_ in a bovine isolate not associated with mastitis is concerning. No ESBL genes were detected in any of the other isolates. Since ESBL are a major threat to human health, monitoring for ESBL genes is required to understand the prevalence among *S. aureus* in cattle to avoid dissemination through cattle production.

There are five major staphylococcal enterotoxins responsible for food poisoning: SEA, SEB, SEC, SED and SEE [65]. Host association for the different *sec* gene subtypes has been described previously, with *sec1-4* being mainly associated with human *S. aureus* isolates and bovine and ovine-specific variants identified [65,66]. Genes from three classes were detected in very few isolates (<15), with the presence of *sea*, *sec2*, *sec3* and *sed* only associated with human isolates in this study. *Sec1* was found in five bovine isolates and one human isolate. Outbreaks of staphylococcal food poisoning have been linked to the consumption of raw milk products, such as soft cheeses, obtained from cattle with mastitis [67,68].

Five virulence genes were conserved in all 96 isolates in this study, of which three were haemolysin genes (*hld*, *hlgA* and *hlgC*) associated with cattle mastitis. Alpha haemolysin is the most common cytotoxin produced by *S. aureus*, which causes a gangrenous type of mastitis in cattle [69]. Beta-haemolysin (hlgB) was found in 100% human and in 72% bovine isolates. The other two genes present in 100% of isolates were *aur*, encoding for aureolysin, a conserved protein involved in the proteolytic processing of other staphylococcal proteins, and *icaC*. The process of biofilm formation is regulated by the intercellular adhesion operon *ica* and the negative regulator *icaR* [70]; however, the *icaA,B,D* or *icaR* genes were not identified in any isolate.

Bi-component leucotoxins are composed of two distinct proteins constituting a family of pore-forming toxins [71]. LukD/LukE were found in 61 and 82% of isolates in this study, respectively. LukM/F, previously reported to be the most cytotoxic leukocidins and associated with gangrenous mastitis [72,73], was not found in any bovine isolate. The human equivalent, Panton and Valentine leukocidin virulence genes encoded by *lukF-PV* and *lukS-PV* [74], were only found in a single human isolate.

Since many *S. aureus* virulence and AMR genes are associated with mobile genetic elements (phages, pathogenicity islands, plasmids and staphylococcal cassette chromosomes) [75], surveillance of non-mastitis-causing isolates is important. Selection pressure by environmental conditions can drive horizontal gene transfer, and along with other members of the microbial community, non-pathogenic *S. aureus* can be the source of virulence/AMR genes.

Since *S. aureus* is an important cause of mastitis in dairy herds, with single strains known to infect multiple cows [18], transmission prevention remains an important tool for the control of mastitis. However, animal–animal transmission is not the only concern; the previous studies have shown transmission of MRSA from cattle to humans or vice versa [76,77]. Environmental or commensal *S. aureus* can also be the source of human infection and are commonly found associated with milking equipment (milking machines, udder cloths or milker’s hands) [18,76].

We investigated asymptomatic *S. aureus* from inguinal skin or nasal carriage in cows and humans to examine potential zoonotic transmission. A total of 14 transmission clusters were detected, with 13 clusters restricted to a single species. Only one transmission cluster suggested zoonotic transmission of an S. *aureus* ST5 isolate from human to animal or vice versa. While the two human ST5 isolates of this cluster were detected in one household, other members of the same household (siblings and parents) did not carry this specific isolate.

All four isolates in the cluster carried an identical ~54-kb φSa3 prophage, a common prophage type associated with >90% of human nasal carriage isolates [78,79], typically harbouring the human immune evasion cluster genes staphylokinase, enterotoxins, chemotaxis-inhibitory protein and staphylococcal complement inhibitor [80]. As these virulence genes are very human-specific, the prophage is typically lost after the host switches from humans to animals [81]. Since both bovine isolates carry φSa3, this might indicate that transmission occurred from humans to cows. However, a low prevalence of these prophages is also found in animal isolates [81]; therefore, the direction of transmission cannot be determined with certainty.

Evidence of transmission among cattle was mostly limited to a single age group, suggesting that transmission occurs within age groups, as different age groups are managed separately on farms. Of five potential transmission clusters that were found for single age groups on a single farm, two were within adult cows during round 1 (mid-milking season), and three were within young calves soon after calving. Milking machines, farmer’s hands or contaminated bedding are all known risk factors for *S. aureus* transmission [3,19]. In the case of cluster 17, the R1s were just recently moved from the newborn pen to the field, so transmission could have occurred in the pen and remains effectively within the same age group.

Of the two clusters involving multiple age groups of cattle on a single farm, one included adult cows and calves soon after calving (cluster 23), suggesting transmission between cows and their calves despite being separated soon after birth. The other cluster (cluster 15) was in both age groups of replacement heifers (those in their first year of life and those in their second) during the second sampling round (dry period) and then moved to the milking herd at calving (when the heifers in their second year are merged into the milker herd), suggesting that merging herds may encourage transmission.

We detected potential transmission between humans less often than between cattle, with four clusters of two human isolates each. The largest cluster with four isolates suggested transmission within a household (children and parents), as well as a potential transmission event at school. These are similar findings to our previous study in another school in New Zealand, where potential transmission was seen between siblings and between small numbers of individuals attending the same school [82]. The detected bovine clusters on average were larger with a minimum of three and up to seven isolates, but given that we sampled a smaller number of humans than cattle and did not sample all cattle, we cannot draw conclusions from the relative size of transmission clusters in cattle and humans and the direction of transmission.

### Study limitations

Sampling methods, effort and conditions may affect the detection of carriage in different species and age groups. While it has been reported that the prevalence of *S. aureus* in the nares was similar to carriage in the inguinal region for healthy bovines [13], there might be differences in skin conditions, e.g. inguinal skin of adult cows during milking may be wetter than inguinal skin of a heifer, and make it either harder or easier to detect true inguinal carriage. Similarly, nasal swabbing of humans may be better or worse at detecting carriage compared with skin swabbing of cattle.

Furthermore, WGS was not performed for all isolates due to financial constraints, and WGS of the additional samples might have detected further evidence of transmission or additional virulence or AMR information. Variable coverage of the reference genome might also have a confounding effect on the transmission results. Another limitation to bear in mind is that genome-predicted AMR results are not necessarily supported by phenotypic testing [83], and vice versa, where phenotypic resistance might be shown, but no known associated mutation can be found [84], highlighting the importance of performing additional antibiotic susceptibility testing in the laboratory. For this study, no additional laboratory analysis was performed, as the focus of the study was zoonotic transmission.

## Conclusion

This research has contributed evidence concerning the frequency and patterns of transmission of *S. aureus* on dairy farms in rural New Zealand. In brief, it has shown that transmission between cattle and humans is less frequently detected than within-species transmission, even though the nature of the work on dairy farms requires close contact between humans and cattle. The study has shown that key transmission points between animals on farms are (1) between animals in the same herd/management group (management groups defined by age on most dairy farms), (2) between cattle and newborn calves and between newborn calves managed together and (3) movement of cattle between management group (e.g. when heifers calving for the first time join the older herd of milking cows). These key transmission points offer insights into where intervention may help to minimize transmission within dairy herds in New Zealand. This study offers means by which to reduce the prevalence of *S. aureus* on dairy farms, which will be useful both for animal welfare and production reasons and to reduce any spillover to human populations.

## supplementary material

10.1099/mgen.0.001318 Uncited Supplementary Material 1.

10.1099/mgen.0.001318 Uncited Supplementary Material 2.

10.1099/mgen.0.001318 Uncited Supplementary Material 3.

## Abbreviations

AMR: antimicrobial resistance
CC: clonal complex
ESBL: extended-spectrum beta-lactamases
LA-MRSA: livestock-associated MRSA
ML: maximum likelihood
MRSA: methicillin-resistant *Staphylococcus aureus*
SCC*mec*: staphylococcal cassette chromosome *mec*
STs: sequence types
WGS: whole-genome sequencing
