# Supplementary material for: Zoonotic transmission of asymptomatic carriage Staphylococcus aureus on dairy farms in Canterbury, New Zealand
Source: Microb Genom. 2024 Dec 4;10(12):001318. doi: 10.1099/mgen.0.001318 (PMC11616781; doi:10.1099/mgen.0.001318)
Supplement: Uncited Supplementary Material 1. [file mgen-10-01318-s001.pdf]

Supplementary Figures

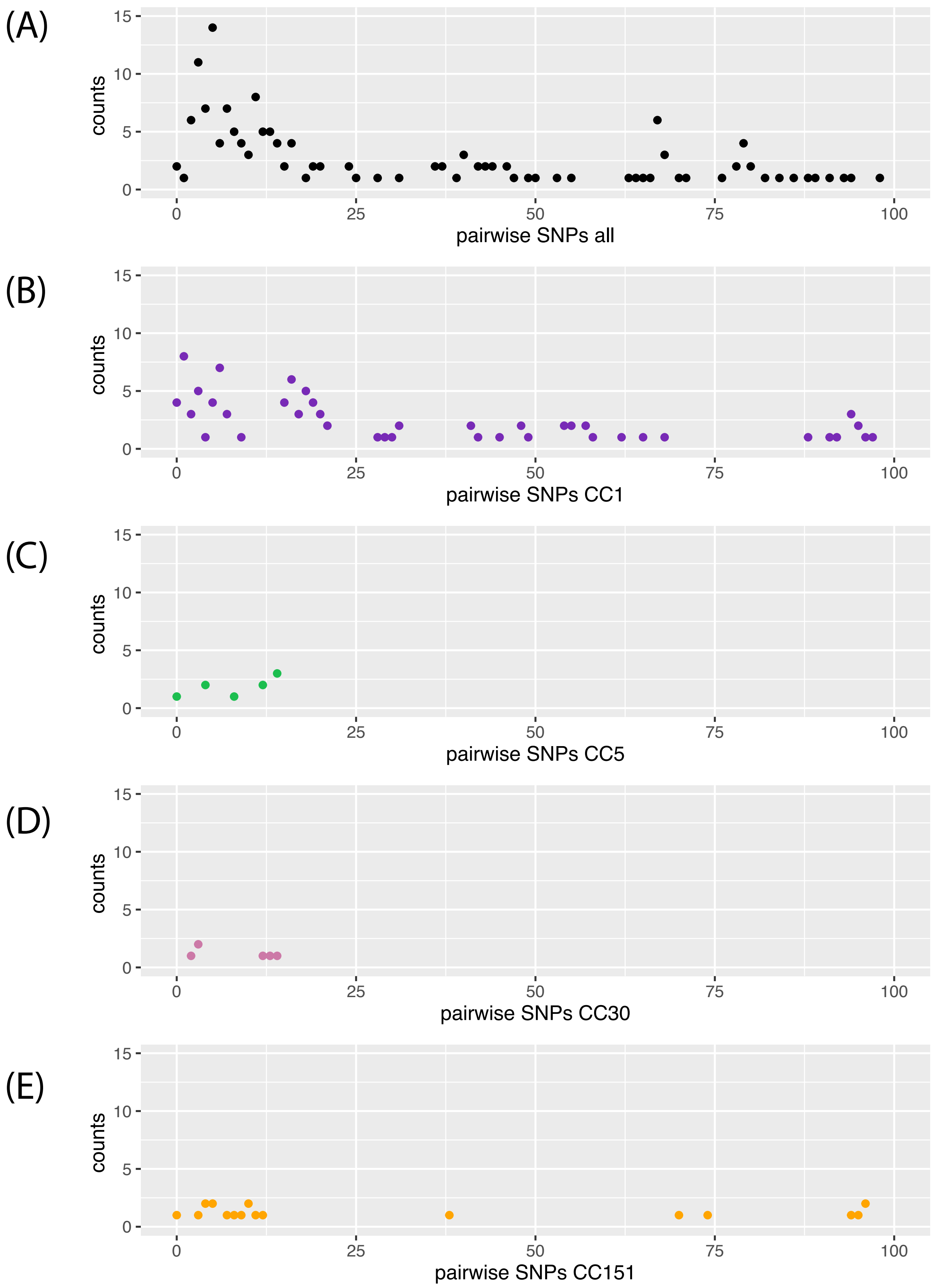

Figure S1. Distribution of pairwise SNP distances among (A) all isolates, (B) CC1, (C) CC5, (D)CC30 and (E) CC151 isolates. The pairwise distance SNP cut-off for plotting was 100 SNPs.

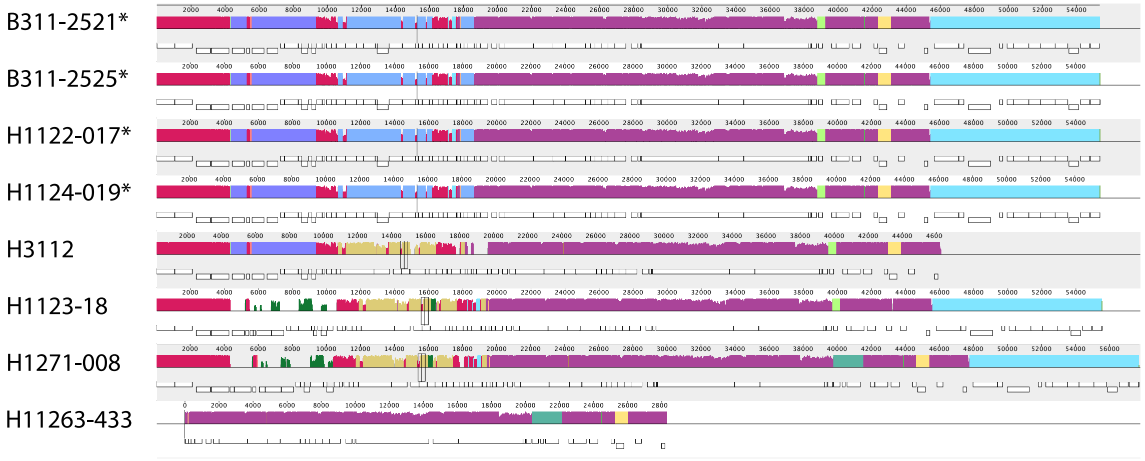

Figure S2: Mauve alignment showing the similarity of the ϕSa3 prophage in all eight ST5 isolates. The isolates belonging to the zoonotic transmission cluster are highlighted with an asterisk.
